# μECoG Recordings Through a Thinned Skull

**DOI:** 10.3389/fnins.2019.01017

**Published:** 2019-10-01

**Authors:** Sarah K. Brodnick, Jared P. Ness, Thomas J. Richner, Sanitta Thongpang, Joseph Novello, Mohammed Hayat, Kevin P. Cheng, Lisa Krugner-Higby, Aaron J. Suminski, Kip A. Ludwig, Justin C. Williams

**Affiliations:** ^1^Department of Biomedical Engineering, University of Wisconsin–Madison, Madison, WI, United States; ^2^Department of Biomedical Engineering, Mahidol University, Salaya, Thailand; ^3^Department of Surgical Sciences, School of Veterinary Medicine, University of Wisconsin–Madison, Madison, WI, United States; ^4^Department of Neurological Surgery, University of Wisconsin–Madison, Madison, WI, United States

**Keywords:** thinned skull, μECoG, local field potenitals, optogenetics, somatosensory evoked potentials

## Abstract

The studies described in this paper for the first time characterize the acute and chronic performance of optically transparent thin-film micro-electrocorticography (μECoG) grids implanted on a thinned skull as both an electrophysiological complement to existing thinned skull preparation for optical recordings/manipulations, and a less invasive alternative to epidural or subdurally placed μECoG arrays. In a longitudinal chronic study, μECoG grids placed on top of a thinned skull maintain impedances comparable to epidurally placed μECoG grids that are stable for periods of at least 1 month. Optogenetic activation of cortex is also reliably demonstrated through the optically transparent μECoG grids acutely placed on the thinned skull. Finally, spatially distinct electrophysiological recordings were evident on μECoG electrodes placed on a thinned skull separated by 500–750 μm, as assessed by stimulation evoked responses using optogenetic activation of cortex as well as invasive and epidermal stimulation of the sciatic and median nerve at chronic time points. Neural signals were collected through a thinned skull in mice and rats, demonstrating potential utility in neuroscience research applications such as *in vivo* imaging and optogenetics.

## Introduction

Electrophysiological recordings of brain activity using high density electrode arrays are a staple of neuroscience research and have become increasingly prevalent for the clinical diagnosis of epileptic seizure foci as well as the clinical deployment of brain–machine interfaces (BMI) (Osorio et al., 2002; Leuthardt et al., 2004, 2006; Viventi et al., 2011). Traditional electrophysiological recording methods involve the implantation of invasive electrode arrays either indwelling within cortex (Kipke et al., 2003; Normann and Fernandez, 2016), beneath the dura (subdural) (Wyler et al., 1984; Henle et al., 2011; Khodagholy et al., 2014), on top of the dura (epidural) (Thongpang et al., 2011; Park et al., 2014; Spuler et al., 2014), or non-invasively on the skin directly above the exterior of the skull (Myrden and Chau, 2015). It is generally accepted that electrode placement closer to the neural signal sources of interest within the brain yields a more information rich and spatially distinct signal (Fernández et al., 2014), whereas activity measured at a distance non-invasively is attenuated in part by the high impedance skull, yielding less spatially distinct information in the recorded signal from electrode to electrode (Grill et al., 2009; Uriguen and Garcia-Zapirain, 2015).

More recently, there has been a growing appreciation that surgical methods to open the skull, and/or the placement of an indwelling electrode grid on or within cortex, may cause adverse effects that impact the neural circuitry of interest (Fernández et al., 2014; Goss-Varley et al., 2017; Falcone et al., 2018). For example, increased glial scarring (Grill et al., 2009; Marin and Fernández, 2010; Salatino et al., 2017), large increases in temperature of cortex (Shoffstall et al., 2018), changes in intracranial pressure (Onal et al., 2003), intracranial hemorrhage and/or physical depression of cortex (Onal et al., 2003; Marin and Fernández, 2010; Degenhart et al., 2016), and bacterial infection (Onal et al., 2003) have all been linked to the surgical procedure and implantation of electrocorticography (ECoG) or indwelling cortical arrays. These adverse events cause subtle changes to the neural circuitry of interest that have been shown to cause long-lasting deficit in performance of fine motor tasks among other consequences (Goss-Varley et al., 2017).

Concurrently there has been an increasing interest in neuroscience experiments that thin the skull instead of removing it, and that use optical methods to record and manipulate both neuronal and non-neuronal cells within the brain (Yang et al., 2010; Shih et al., 2012; Bonder and McCarthy, 2014). Removal of the skull in rodents has been shown to create glial scarring in the area under the craniotomy (Yang et al., 2010). Unlike the outer compact layer of the skull which has low conductivity, the spongy bone of the skull closer to the brain is low impedance (Akhtari et al., 2002) and if thinned appropriately is optically transparent (Drew et al., 2010). However, the performance of μECoG grids placed chronically on a thinned skull preparation has yet to be evaluated.

To address this gap, a series of acute and chronic studies was performed where the skull was thinned to a translucent layer and implanted with a μECoG electrode array. μECoG arrays were used in the experiments described because of their flexibility, transparency, and well-characterized epidural signal profile (Thongpang et al., 2011; Park et al., 2014). In rats chronically implanted for 1 month, impedance values and somatosensory evoked potentials (SSEPs) were recorded at regular intervals to assess stability of electrical function and spatial resolution of recordings through the thinned skull. Cortical signals from optogenetic stimulation in a ChR2 mouse were recorded in an acute terminal session through a thinned skull and were compared to recordings through the dura after removal of the thinned skull. These studies tested multiple common stimulation paradigms for neuroscience research in multiple species, mice, and rats, to characterize the reliability and spatial resolution of electrophysiological recordings through a thinned skull.

## Materials and Methods

### Ethics Statement

All animal procedures were approved by the Institutional Animal Care and Use Committee (IACUC) at the University of Wisconsin–Madison, Madison, WI, United States. All efforts were made to minimize animal discomfort.

### Device Fabrication

**FIGURE 1:**
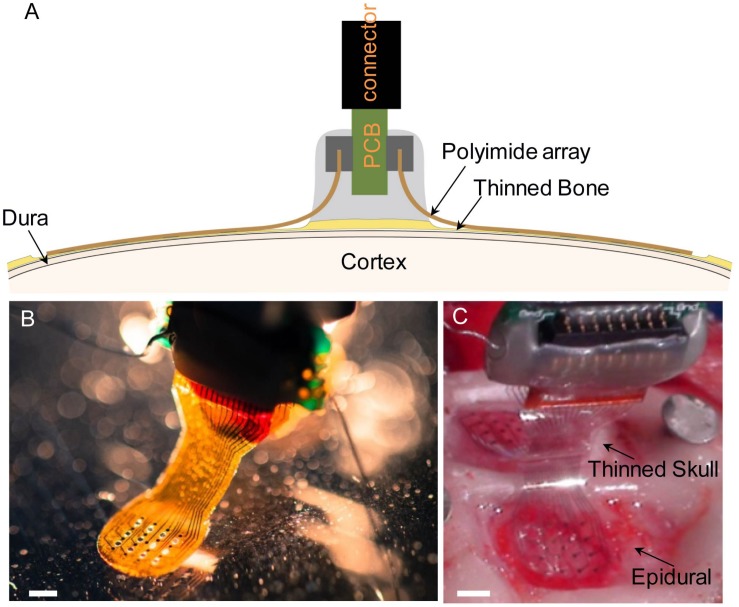
**(A)** Diagram illustrating chronic placement of 16-channel bilateral μECoG array, and ZIF connector on thinned skull surface over sensorimotor cortex in a rat. **(B)** Polyimide-based platinum μECoG array with 16 channels (750 μm spacing, 250 μm site diameter). **(C)** Surgical photograph of bilateral parylene C-based platinum μECoG array being placed over a thinned skull (top of photograph) and on the dural surface (bottom of photograph). Scale bars in panels **(B,C)** represent 2 mm.

**FIGURE 2:**
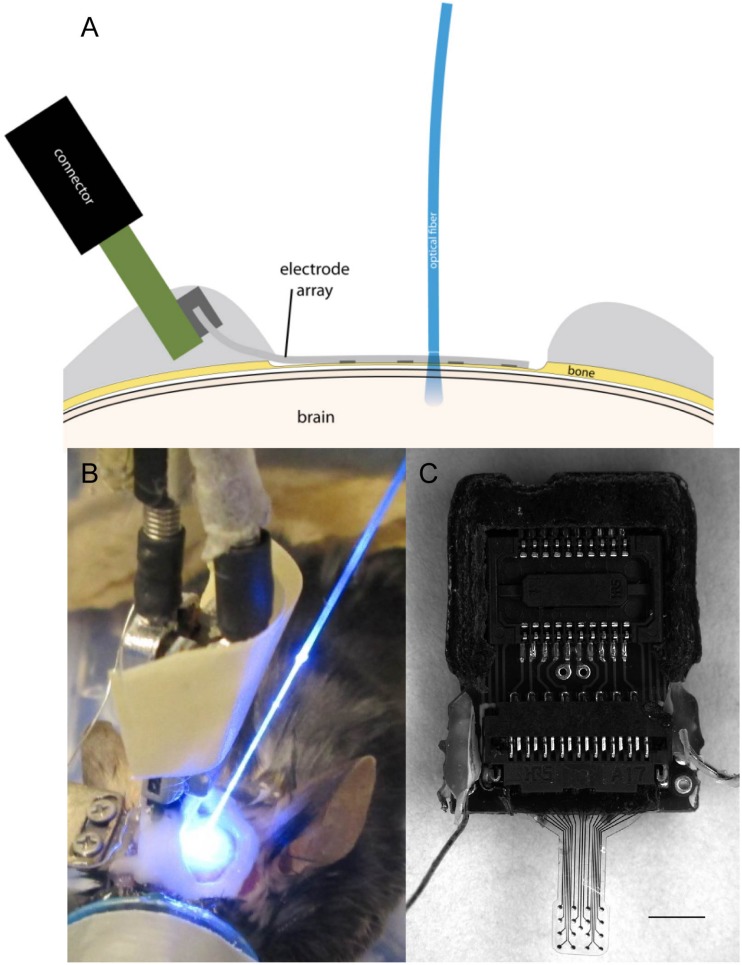
**(A)** Illustration of μECoG electrode array placement over a thinned skull in a mouse with optical fiber positioning. **(B)** Optogenetic stimulation of cortex with optical fiber placed on a μECoG array over thinned skull. **(C)** Parylene C-based platinum μECoG array with 16 channels (500 μm spacing, 150 μm site diameter) and ZIF connector. Scale bar in panel **(C)** represents 2 mm.

Micro-electrocorticography array advanced measurement testing has been published previously and validated to record neural signals (Thongpang et al., 2011; Park et al., 2014; Richner et al., 2014). μECoG devices were fabricated following protocols previously described for polyimide (Thongpang et al., 2011) and parylene C (Schendel et al., 2013) arrays. Briefly, photodefinable polyimide was used to pattern polyimide and a chemical vapor deposition system was used to pattern parylene C onto silicon wafers. Photolithography, metal deposition (Cr/Au/Pt), and lift off and plasma etching allowed for patterning of the electrodes, traces, and array shape. Final thickness of arrays was 25 μm. Rat sized polyimide (spacing between recording sites: 750 μm, 250 μm site diameter) (Figures 1A,B) or parylene C (spacing between recording sites: 750 μm, 200 μm site diameter) (Figure 1C)-based μECoG electrode arrays were custom fabricated with 16 platinum sites (one or two 4 mm × 4 mm grids) and implanted unilaterally or bilaterally between bregma and lambda in Sprague-Dawley rats. Similarly, for experiments with mice, a smaller, 2 mm × 2 mm 16 platinum site parylene-C μECoG array (500 μm spacing, 150 μm site diameter) (Figure 2) was fabricated and used for optogenetic experiments (Figures 2A,B). Parylene C was chosen for optogenetic and imaging studies due to its flexibility and translucent properties (Figure 2C). A density of 16 electrode sites was chosen along with specific interelectrode site spacings in order to visualize the somatosensory cortex forelimb and hindlimb areas, and to visualize where signals were no longer recorded on the periphery of the array (Sakatani et al., 1990). Extensive bench top testing of μECoG arrays has been preformed in previous publications to validate neural signal recordings.

### Surgical Preparation

#### Chronic Experiments

Male Sprague-Dawley rats (*n* = 7, Envigo, Indianapolis, IN, United States) 2–4 months old were chronically implanted with custom built polyimide or parylene C μECoG arrays with metal electrodes (Figure 1A). Three rats were implanted with bilateral arrays over thinned skull, three rats were implanted with bilateral arrays over the dural surface, and one rat was implanted with a bilateral array, one over thinned skull and one over the dura. The electrode array on the dural hemisphere for this animal was damaged during implantation and not viable therefore electrophysiology and impedance data were not included in this paper; however, histological staining was performed and included. One possible failure mechanism may have been that the flat-flex connector between the array and PCB resulted in the signals of the epidural array being an open circuit and therefore noisy. Surgical procedures were based on previously published methods (Park et al., 2016). Before surgery, buprenorphine hydrochloride (0.05 mg kg^–1^, Reckitt Benckiser Healthcare) was administered for analgesia and dexamethasone (2 mg kg^–1^, AgriLabs) to prevent cerebral edema. Rats were induced with 5% isoflurane gas in O_2_ and maintained on 1.0–2.5% throughout the duration of the surgery. Following induction, rats were placed into a stereotaxic frame with the scalp shaved and prepped with alternating povidone iodine and alcohol. The skin was incised, and the exposed skull was cleaned and dried. Three stainless steel screws (stainless steel, 00–80 × 1/8 inch), two for attachment of a ground wire, and another for reference and mechanical support, were attached to the rostral and caudal areas of the skull. Next, UV curable dental acrylic (Fusio, Pentron Clinical) was placed on the periphery of the exposed skull to provide an anchor for the attachment of future acrylic, and two craniotomies (∼5 mm × 5 mm) or thin skull areas were drilled over somatosensory cortex. A thinned skull area was made by drilling through the top layer of compact bone, through the spongy layer, and slightly into the lower compact bone where it became transparent. We estimate the final thickness of the lower compact bone layer as ∼100–200 μm as the adult rat skull at 90 days is ∼700 μm thick (Gefen et al., 2004). #106 and #107 spherical drill burrs were used. Saline irrigation was used to remove debris and to lessen the effect of heating from the drill. The μECoG arrays were placed epidurally or over a thinned skull area and covered with a thin layer of GelFoam (Pharmacia and Upjohn Co., New York, NY, United States) and saline before being covered by dental acrylic. GelFoam was used to prevent acrylic from covering the electrode array and was only placed on top of the arrays and not beneath. The ZIF connector was then secured to the skull and a purse string suture (3–0 vicryl) closed the skin wound. Triple antibiotic ointment was applied to the wound during closing to prevent infection. Rats were monitored post-surgically until they were ambulatory and showed no signs of pain or distress. Another dose of buprenorphine was administered 8–12 h after the initial dose to relieve any pain the animal may have been experiencing following the surgery. Ampicillin [50 mg kg^–1^ subcutaneous injection (SC), Sage Pharmaceutical] was administered twice daily for 7 days postoperatively to prevent infection.

#### Acute Terminal Experiments

Three Thy1::ChR2/H134R-YFP (ChR2) mice (Jackson Laboratory; stock number 012350) ∼6–16 weeks old were implanted during acute terminal recording sessions with clear parylene C μECoG arrays implanted over the dura or a thinned skull area to compare neural signals recorded from light stimulation (Figures 2A,B). Evoked potential data from optogenetic stimulation were collected from three mice and strength duration curve data were collected from one mouse. Arrays were placed onto a thinned skull first, and then placed epidurally after removing the thinned skull. Mouse surgical procedures were similar to previously published methods (Richner et al., 2014). Briefly, mice were administered buprenorphine hydrochloride (0.05 mg kg^–1^) and dexamethasone (1 mg kg^–1^ SC) before induction, induced, and maintained with 1–2.5% isoflurane. The animal was placed in a stereotaxic-like frame and a craniotomy or thinned skull was performed. A μECoG array was placed on the dura or thinned skull and ground and reference wires were coiled and placed on a small area of thinned skull on the contralateral hemisphere. The skull was thinned in the mice to the lower compact bone similar to the rat but using #105 and #106 spherical drill burrs. After drilling the skull was optically transparent and thickness was ∼50 μm or thinner (Shih et al., 2012). GelFoam was not used in optogenetic studies. Instead the cortical surface was continually kept wet with a saline drip.

Heart rate and blood oxygen concentration in both species were monitored throughout the surgery using a pulse oximeter. Body temperature was monitored with a digital thermometer and regulated with a water-circulated heating blanket.

### Electrophysiological Testing

#### Periodic Chronic Electrophysiology Testing

Sensorimotor evoked potentials were recorded periodically for up to 1 month under sedation in rats with chronic μECoG implants to assess signal stability and uniqueness/spatial resolution of information recorded on nearby sites. Dexmedetomidine (50 μg/kg SC) was used to achieve sedation. Atipamezole (0.5 mg/kg SC) was administered at the end of the procedure as a reversal agent. Dexmedetomidine sedation was supplemented with small amounts of isoflurane (0–0.5%) throughout the procedure to deepen sedation. The sciatic or median nerve, hindlimb or forelimb, respectively, were stimulated weekly to evoke SSEPs. Needle or surface stimulation electrodes were used. Needle electrodes were placed on either side of the sciatic nerve, 3 mm apart. Surface electrodes were placed on shaved skin above the sciatic or median nerve, with a reference electrode placed below the leg. Stimulation pulses were applied using needle electrodes (monophasic 0–0.8 mA for 2 ms), or surface electrodes (monophasic 0.5–3.5 mA for 1 ms) both at approximately 0.5 Hz. The cortical responses were recorded and digitized simultaneously at 3 kHz using a PZ2 Preamplifier and a RZ2 BioAmp Processor (Tucker-Davis Technologies, Alachua, FL, United States).

#### Acute Terminal Optogenetic Electrophysiological Testing

Optogenetically evoked potentials were recorded during a terminal procedure by shining light through a fiber-coupled LASER system or LED through an optically transparent parylene μECoG onto the dura or thinned skull of ChR2 mice using previously reported methods (Uriguen and Garcia-Zapirain, 2015; Figure 2). Photostimulation was accomplished by using an optical fiber (200 μm in diameter, 0.22 NA, flat cleaved, and polished, Thorlabs, Newton, NJ, United States) connected to a 100 mW 473 nm LASER (Laserglow, Toronto, ON, Canada) and controlled by a multichannel system (TDT, Alachua, FL, United States). 2.5 ms pulses, varying power settings, and random interstimulus intervals were used. Power 1 mm from the tip of the optical fiber was approximately 80 mW/mm^2^. The optical fiber was placed approximately 1 mm from the cortical or thinned skull surface. 3 kHz recordings were obtained and digitized using a PZ2 Preamplifier and a RZ2 BioAmp Processor (Tucker-Davis Technologies, Alachua, FL, United States), and sampled with a high impedance headstage. A photostimulus delivered by an LED (465 nm, RGB MC-E, Cree, Durham, NC, United States) approximately 2 cm away from the cortical or thinned skull surface was used to create photostimulus duration vs. amplitude peak to peak potential contour plots (Figure 7). Voltage pulses were changed to current pulses (0–1000 mA, 0.5–12 ms) with an LED driver (BuckBlock, LEDdynamics, Randolph, VT, United States). Irradiance was calculated by measuring optical power (PM100D, S130C, Thor Labs, Newton, NJ, United States) 2 cm from the LED, and the result was divided by the commercially available photo sensor’s area (S370 Optometer, United Detector Technology, Hawthorne, CA, United States).

### Electrophysiology Analysis

**FIGURE 3:**
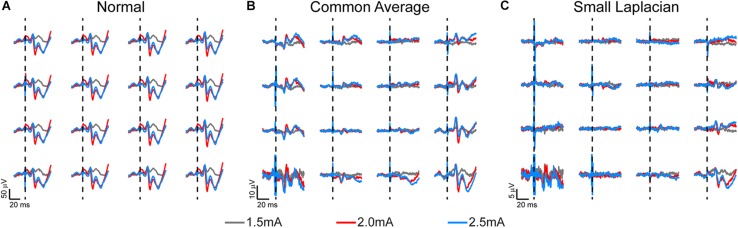
Somatosensory evoked potentials (SSEPs) recorded on week 3 post-implantation from a rat implanted with a 16-channel μECoG array placed on thinned skull over left sensorimotor cortex. Biphasic current pulses (1 ms, varied amplitude) were used to stimulate the right hindlimb with surface electrodes over the sciatic nerve. **(A)** Stainless-steel bone screw, **(B)** common average, and **(C)** small Laplacian referencing strategies are shown to increase the signal-to-noise ratio, and to reveal spatial signaling from the predicted hindlimb anatomical region. Dashed lines represent onset of electrical stimulus.

**FIGURE 4:**
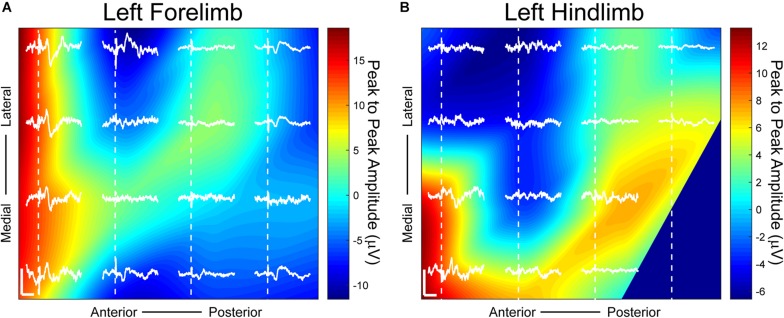
Somatosensory evoked potentials (SSEPs) on day 38 post-implantation with small Laplacian referencing from forelimb and hindlimb electrical surface stimulation using a 16-channel μECoG array placed over a thinned skull portion of rat sensorimotor cortex. Plots represent spatial recordings from the same electrode array, demonstrating LFPs from **(A)** biphasic forelimb stimulation and **(B)** monophasic hindlimb stimulation. Stimuli were applied for 1ms at 1.25 mA. Activity is represented by 2D interpolated heat maps. The portions closer to the red spectrum show evoked activity higher than baseline when averaged over at least 25 trials, and closer to blue shows negative activity. The *x* scale bar, 20 ms; *y* scale bar, 20 μV. Dashed lines represent onset of electrical stimulus.

To examine the efficacy of the evoked responses through the thin skull prep, local-field potentials (LFPs) obtained from varying stimulation, both optogenetic and electric, were analyzed and checked against epidural implants. LFPs were band limited using a combination of a second order, Butterworth lowpass filter (cutoff frequency = 1000 Hz), a Butterworth high pass filter (cutoff frequency = 3Hz), and a third order notch filter (cutoff frequencies = 55 and 65 Hz) to remove line noise. Evoked potentials were computed from the average of evoked responses from the same stimulus amplitude and channel. Positive signal amplitudes reflected positive voltages. To increase signal-to-noise ratios, two known post processing referencing techniques, common average referencing (CAR) and small Laplacian referencing, were employed and compared (Figure 3). Each were incorporated as described in the literature (McFarland et al., 1997; Ludwig et al., 2009). Heatmaps were created to describe the spatial organization of the cortical activity based on the small Laplacian referencing technique (Figures 4, 7). Each heatmap was obtained by taking the maximum peak to peak SSEP from each μECoG electrode. Peaks were defined as the average of seven data points centered on the maximums of the response from a given response window (latency of 10–35 ms). Responses were interpolated using a cubic interpolation algorithm (Matlab griddata) to a meshgrid with 1 μm resolution (Sakatani et al., 1990; Bazley et al., 2012). Peak latencies were measured as the time from the onset of the stimulus to the first contralateral potential peak. We manually selected the peak using a custom GUI in Matlab.

Channels above 600 kΩ were considered to be non-functional and removed from analysis. For the 2 day map interpolation plot (Figure 7), 30 pulse shapes with varying width and duration were applied with interleaved trials. Each pulse shape was repeated at least 20 times. The averaged optogenetic evoked potentials were calculated for each pulse shape and the peak amplitude was measured. The contour plots were then interpolated to find isopotential lines.

### Chronic Impedance Recordings and Analysis

**FIGURE 5:**
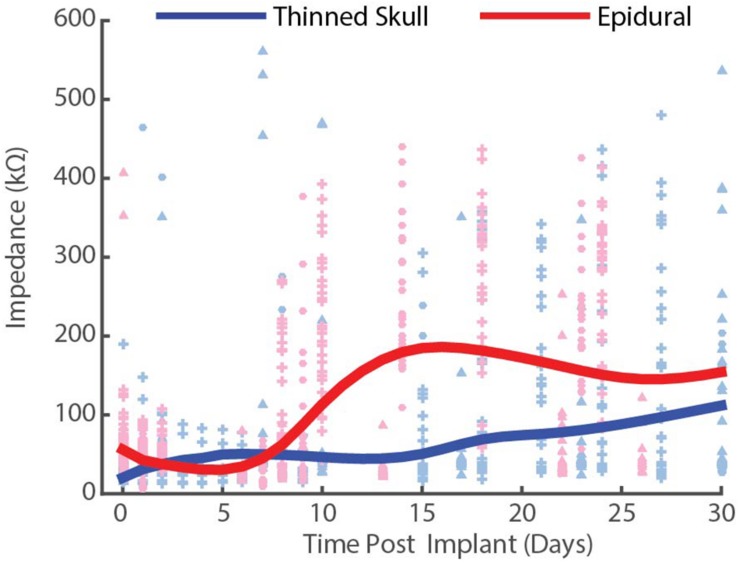
Chronic impedance spectral data at 1 kHz from thinned skull (blue) and epidurally (red) implanted μECoG electrodes in rats. Each interpolation curve represents three animals per group, and 32 electrode sites per animal. Individual data points represent individual electrode site impedance spectra measurements. Each shape represents an individual animal. Epidural impedances increase until ∼2 weeks and plateau, while thinned skull impedances remain lower and more stable.

Electrical impedance spectra were collected from arrays before implantation, and periodically after implantation to assess electrical characteristics using a potentiostat (Autolab PGSTAT 128N, Metrohm, Riverview, FL, United States) and following previously published methods (Figure 5; Williams et al., 2007). Arrays that were determined viable for implantation had values of approximately 50–100 kΩ at 1 kHz. Animals were trained to sit still with treats and were not anesthetized or sedated for chronic impedance measurements. Analysis consisted of data from six rats, three with thinned skull implants and three with epidural implants for comparison. Each rat had a bilateral implant consisting of 32 electrode sites. Impedance measurements were gathered from each electrode for 30 days post implantation. Resistive values at 1 kHz were plotted for each of the 32 channels corresponding to length of time of the implant. Single channels with a resistance >600 kΩ were considered outliers and eliminated from calculations for that day. Outliers were considered to be broken or due to an inadequate connection. Average resistance was plotted for each day and fitted to a curve across days using cubic spline interpolation to account for measurements potentially not lining up exactly on individual days across animals. The thinned skull implants interpolation curves were averaged together and plotted against the epidural implants averaged interpolation curves. Impedance values were not recorded after implantation in acute mice experiments, although pre-implantation impedance values were comparable to those of the devices implanted for chronic recordings in rats.

## Results and Discussion

### Chronic Periodic Sensory Evoked Potential Recordings in Rats

Chronic SSEP recordings were obtained weekly during electrical stimulation of the sciatic or median nerve in three rats to compare the spatial resolution of thinned skull μECoG arrays vs. traditional epidural arrays. Thinned skull μECoG arrays were implanted bilaterally in sensorimotor cortex in each rat and SSEPs were recorded in each contralateral hemisphere from a cutaneous electrical stimulus of hindlimb or forelimb. A representative plot of thinned skull SSEPs on the left hemisphere from right hindlimb stimulation is shown in Figure 3. Using the stainless-steel bone screw as a reference, which was implanted cranial and contralateral to the μECoG array (Figure 3A), recorded signals contained common noise, and differences in SSEPs from nearby electrode locations were not readily apparent. Two post process referencing techniques were used to reduce both common noise and common signal to highlight spatially distinct differences in neural signals. Employing a common average reference (CAR) (Figure 3B) successfully recovered spatially distinct hindlimb SSEPs on adjacent electrode sites. Similarly, employing a small Laplacian (Figure 3C) reference *post hoc* further highlighted spatially distinct SSEP responses on adjacent sites. Consequently, we chose to use the small Laplacian *post hoc* referencing strategy for the remainder of the recording data, because it visually increased unique highlighted spatial information present in the SSEP on adjacent sites (McFarland et al., 1997). Epidural signals are similarly plotted in Supplementary Figure S1, although they are recorded at slightly different post-operative time periods and use different stimulation amplitudes.

Distinct somatotopic signals were recorded 38 days post-implantation from μECoG arrays placed on a thinned skull area of the rat’s right sensorimotor cortex from both left hindlimb and forelimb stimulation (Figure 4). Small Laplacian referencing methods were also applied. Highest peak to peak SSEP values from forelimb stimulation, according to the heatmap, are positioned at the anterior portion of the electrode with peaks spanning both medially and laterally (Figure 4A). When switching the area of stimulation to the hindlimb, SSEPs shifted medially similar to previously mapped rat sensorimotor cortex (Figure 4B; Sakatani et al., 1990). The recorded sensory responses are consistent with the response latencies for myelinated sensory fiber conduction, around 13 ms for forelimb and 17 ms for hindlimb according to previously published data (Sakatani et al., 1990; Bazley et al., 2012).

Thinned skull μECoG electrode arrays not surprisingly have lower signal amplitudes recorded during evoked responses in comparison to historical studies using the same arrays placed epidurally which are closer to the source of the neural signal (Park et al., 2014). As a result, it becomes more important to employ CAR and small Laplacian referencing strategies to eliminate common signal/noise to uncover spatially distinct spatial information for neuroscience applications. Given a similar SSEP was recorded across all electrode sites with appropriate conduction latency prior to *post hoc* referencing, this may suggest the common signal was recorded at the stainless-steel bone screws in contact with the surface of the brain used for the reference and ground, respectively. This referencing strategy was utilized because the ECoG signal recorded from the bone screws has historically been insignificant compared to signals recorded epidurally from μECoG. Therefore, in previous studies *post hoc* referencing was not required to reveal spatially distinct information from site to site. The attenuation of signal through the thin skull in studies described here made the small common signal putatively recorded from the stainless-steel screws more problematic. Consequently, *post hoc* referencing was necessary before spatially distinct SSEPs were observed on adjacent electrode sites.

Thinned skull μECoG electrode arrays also have been shown in this study to record information on a temporal scale similar to epidurally placed arrays. At the relatively low frequencies found in electrophysiological signals, the temporal resistive-capacitive filtering of the bone under the array is minimal. For example, the timing of the SSEP peaks were not appreciably delayed when compared to epidural. Currently, GCaMP6f is a popular genetically coded calcium indication (GECI) that is commonly used to observe neural activity at an onset of approximately 45 ms (Wang et al., 2019). This suggests that the incorporation of an optically transparent μECoG array with common thinned skull experiments for optical imaging would provide unique, complementary temporal information.

### Chronic Periodic Impedance Spectra Recordings in Rats

To compare the electrical performance of epidural vs. thinned skull placed electrodes in rats over time, we measured the impedance spectra of electrodes on each array at 1 kHz periodically over the chronic implantation period (Figure 5). Impedance plots from μECoG arrays implanted on a thinned skull preparation showed slightly different patterns of change over time than those implanted epidurally (Figure 5). Initial electrode impedances were similar when measured in 0.9% w/v phosphate-buffered NaCl saline (∼25–125 mΩ at 1 KHz). After approximately 14 days of implantation as shown in Figure 5, the impedances of the electrodes on the epidural surface were higher on average than that of the electrodes on the thinned skull surface. The epidural impedance interpolation curve shows rise in impedance around 1 week after implantation and lasting for approximately 14 days, similar to other microelectrodes implanted in or on cortex in other studies (Ludwig et al., 2006; Williams et al., 2007). This may be attributed to a central nervous system immune response and new tissue formation and follows previous intracortical and epidural implantation impedance results (Williams et al., 2007; Park et al., 2014). Impedances of epidural implants reached a steady state between 2 and 4 weeks post-implant reflecting decelerated wound healing. In contrast, the thinned skull impedance interpolation curve remained relatively stable for the 30-day period, slowly rising throughout. The difference in shape of the impedance curves suggests the chronic thinned skull electrode/tissue interface is slightly different in composition than the epidural grids. Even though the curve shapes differed, the beginning and end point of data collection (days 0 and 30) between the thinned skull and epidural groups were similar (within ∼50 kΩ of each other). This demonstrates that bone regrowth/scarring under the thinned skull electrodes does not grossly increase the impedance by comparison. Supplementary Figure S2 shows line plots of individually recorded impedance values from each rat over a time period of 1 month.

Decreased impedances during the first few weeks of thinned skull electrode implantation may suggest edema, and that fluid remained at the electrode/tissue interface without clearing. Extra fluid could have hypothetically caused shunting of current and increased distance between the electrode array and the thinned skull. Regardless, we were still able to record spatially and temporally accurate SSEPs and optogenetically induced field potentials from thinned skull electrodes in rats and mice with relatively low impedance values (impedance values not acquired post-implantation in acute mice studies).

### Comparison of Thinned Skull vs. Epidural Recordings From Optogenetic Light Stimulation in Acute Terminal Mice

**FIGURE 6:**
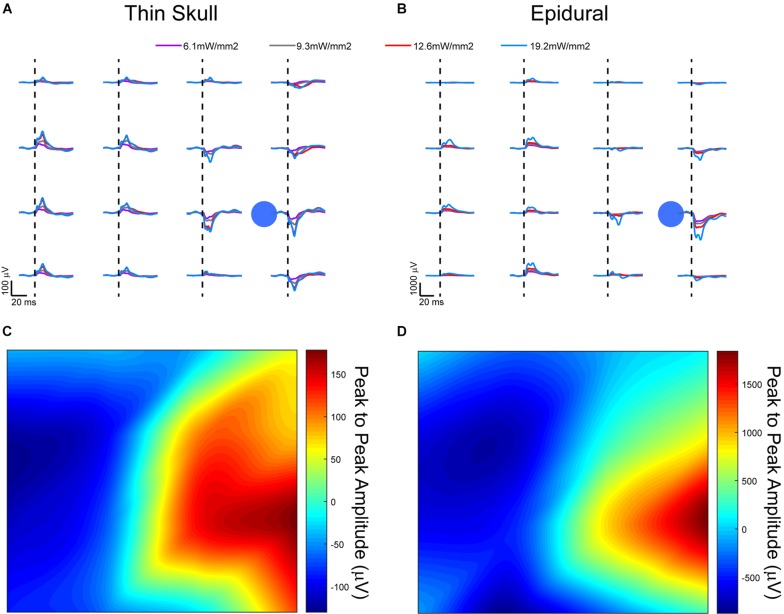
Optically evoked local field potentials from **(A)** thinned skull and **(B)** epidurally implanted μECoG arrays in an acute, terminal Thy1-ChR2 mouse. Amplitude heat maps show the 545.5 mW/mm^2^ optically evoked potentials using small Laplacian referencing from both the **(C)** thin skull and **(D)** epidural preparations. Each can be processed to illustrate the spatial resolution of the recordings, although the difference in scale is smaller in the thinned skull preparation by approximately a magnitude of 10. Dashed lines represent onset of electrical stimulus.

To further investigate the spatial resolution of information on nearby electrodes given a thinned skull recording approach, a light stimulus was applied through a clear parylene C μECoG array and thinned skull to optogenetically activate neurons expressing light sensitive proteins. Optogenetically evoked potentials were recorded in ChR2 mice through a thinned skull (Figure 6A) and epidurally using a smaller 2 mm × 2 mm clear μECoG array to generate a consistent focal activation of cortex for comparison. Even though the array’s substrate (parylene C) was clear, the metal electrodes and traces were not and blocked some portions of light. All optogenetic experiments used the same type of devices and had the same amount of light loss from metal. Using clear electrode sites and traces made of materials like In tin oxide, ITO, or graphene may help alleviate this problem in the future.

A comparison of signals from these two groups did present some confounds. Most likely there was a different scattering of light that arrived at the cortex in the thinned skull vs. the epidural cohorts. While quantifying this scattering of light through the thinned skull would be interesting, it would not be trivial to measure or model the light exiting the skull in each individual animal since the thinning technique which drills to the most transparent bone layer leaves the bone not completely uniform in thickness. Also, we wanted to demonstrate that even without an exact measurement of uniform bone, a simple thinning procedure could be performed to record useful neural signals.

Evoked responses through the thinned skull showed the highest peak responses near the foci of optogenetic stimulation after small Laplacian referencing, further demonstrating the spatial recording ability of the preparation (Figure 6A). Increasing 473 nm laser power also increased evoked potential peak amplitudes. A similar response paradigm occurs with epidural stimulation and recording (Figure 6B), however, we obtain a much larger signal possibly due to lack of spatial filtration of signal through the skull. The additional layer of thin bone also caused additional scattering of the blue light before it reached cortex, slightly changing the optogenetic stimulus between the two conditions. Figures 6C,D use a 2D interpolated heat map to show differences in peak to peak amplitudes at a stimulation laser power of 545.5 mW/mm^2^. Both thinned skull and epidural heat maps display spatial distinct recordings on nearby electrode sites. Due to the presumed filtration/attenuation of signal through the skull and other tissues, the thinned skull recording (Figure 6C) is approximately 10 times less in peak-to-peak amplitude than the epidural recording (Figure 6D). The thinned skull signal also seems to be slightly more diffuse given appropriate referencing strategies. This may also be due to the scattering of light through the skull. Future studies could test different electrode diameter sizing and inter-site spacing to better specify the spatial properties of the signals recorded through a thinned skull.

**FIGURE 7:**
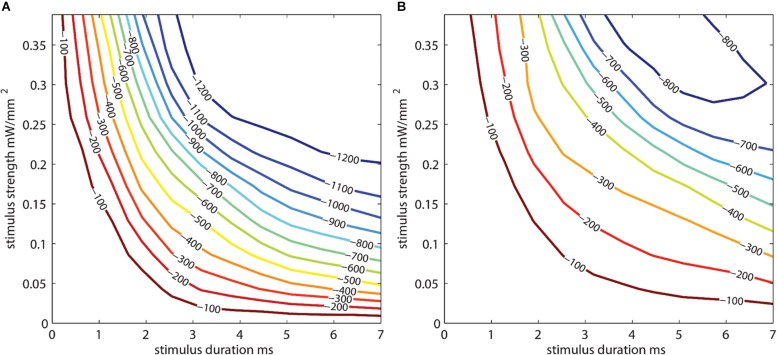
Photostimulus duration vs. amplitude peak potential 2D interpolated contour plot. Stimulus strength is plotted against stimulus duration. Interpolated curves denoting the peak depolarization amplitudes (in μV) for the stimulus strength/duration are shown for **(A)** epidural and **(B)** thinned skull μECoG recordings in an acute terminal Thy1-ChR2 mouse. Longer stimulus durations and stimulus strength (power) are needed to evoke similar sized neural signal amplitudes in the thinned skull vs. epidural preparations. The stainless-steel skull screw reference was used for this analysis. No additional software referencing techniques were used.

One ChR2 mouse underwent an acute procedure where the skull was thinned, and a 465 nm LED was positioned 2 cm away from the thinned skull with the light power and duration values varied to generate photostimulus strength vs. duration curves (Figure 7). The resulting illumination covered most of the cortical area under the μECoG array. Stimuli strength and duration were applied randomly, and peak amplitudes of signals recorded. The thinned skull was then removed exposing the dural surface and stimulation procedure repeated. Figure 7A depicts a contour plots for signals recorded from the dura, whereas Figure 7B depicts the same plot from the thinned skull. The stainless-steel skull screw reference was used for Figure 7 analysis. No additional software referencing techniques were used.

The optogenetically evoked μECoG signal on both the epidural and thinned skull grids demonstrated spatially distinct information, with waveform reversals often apparent on two adjacent sites. These reversals, in conjunction with the waveshape of the evoked response, demonstrated that the electrophysiological recordings were not photoelectric artifacts. Although the magnitude in μVolts of the evoked signal was approximately 10× less with the thinned skull preparation than with the epidurally placed grids, the spatial information as assessed by differences in recordings at adjacent electrode sites was highly similar after *post hoc* small Laplacian referencing.

### Imaging of Immune Response in Neural Tissue to Thinned Skull and Epidural μECoG Implantations

Given the impedance responses over time in all animals, histology was performed on rat M32 to compare histology to the impedance measurement of approximately 50 kΩ at timepoint 32 days post-implantation. Histologic sectioning and immunochemical staining for glial fibrillary acidic protein (GFAP) for astrocytes and Iba-1 for microglia and/or infiltrating macrophages were performed with perfused neural tissue in the single rat with bilateral thinned skull/epidural μECoG arrays (Supplementary Figure S3). The cortical region directly beneath the epidural preparation showed putative increase in GFAP immunoreactivity and projection of astrocytic processes toward the cortical surface (Supplementary Figure S3C). Iba-1 staining did not reveal any obvious increases in microglial immunoreactivity within the brain tissue on either the epidural or thinned skull hemispheres (Supplementary Figure S3D). However, an apparent thickening of the dura on the epidural side was observed (Supplementary Figure S3F) which contained a higher density of Iba-1-positive cells, either microglia or infiltrating macrophages, that was not present on the thinned skull side of the animal (Supplementary Figure S3E). Previous studies have also reported thickening of the dura under the μECoG array consisting primarily of collagen (Schendel et al., 2014; Degenhart et al., 2016).

The main benefit of the thinned skull preparation is that the skull remains partially intact. When the skull is completely removed many side effects can occur which may impact the interpretation of behavioral results. Previous studies in the field of *in vivo* imaging have showed increased glial reaction (microglia and astrocytes) under an open craniotomy window preparation compared to a thinned skull window preparation in mice (Yang et al., 2010). Pneumocephalus can occur after craniotomy in a clinical setting which involves air being trapped in the cranial cavity (Reasoner et al., 1994). Also, dendritic spine plasticity has been shown to differ in thinned vs. open-skull window preparations, emphasizing that the neural environment under a craniotomy may be changed by the craniotomy itself (Xu et al., 2007; Yang et al., 2010). Another benefit the thinned skull recording technique might offer is improved implant mechanical stability, and the lack of direct contact with the surface of the brain or dura. The latter may reduce the risk of injury to neural tissue or device failure due to the lack of device movement on the surface of the brain, although this will need to be investigated in further studies.

## Conclusion

In summary, the studies described in this paper for the first time demonstrate that μECoG grids placed on a thinned skull can provide stable, spatially distinct electrophysiological information out to periods in excess of a month in rats. Mice implanted with a clear polymer μECoG array allowed for simultaneous electrophysiology and optical access through a thinned skull, although these studies were performed acutely. Chronic studies of these type would need to be performed to ensure similar results. Our chronic thinned skull neural recordings in conjunction with previously published chronic thinned skull window imaging (Shih et al., 2019) suggests that collecting these chronic data may be possible.

Neural recordings through a thinned skull is complementary to optical imaging techniques in a number of ways. First and perhaps foremost, ECoG is an established clinically viable diagnostic and therapeutic technique in human patients (Tripathi et al., 2010). The ability to combine optogenetics and optical recordings with μECoG allows for one to better understand and optimize the clinically viable ECoG system. Secondly, optical imaging is limited by the temporal resolution of the fluorescent indicator. This makes it difficult to infer number of synapses involved when a specific pathway is activated, such as is commonly done in SSEPs. Moreover, many studies look at pathological oscillations at higher frequencies, such as up to 30 Hz for tremor (Schnitzler and Gross, 2005), where the temporal resolution of the optical indicator becomes a potential confound due to undersampling the intrinsic oscillation. μECoG serves as a complement to more accurately measure the frequency component of intrinsic oscillations. Finally, unlike optical recording techniques that measure from superficial regions of cortex, ECoG is known to record activity originating from deeper areas of the brain (Ojemann et al., 2013; Richner et al., 2019). Future improvements to this method would include using optically transparent electrode sites and traces made out of transparent metals or other materials such as graphene. This would allow for complete light transmission through the μECoG array.

The ability to record neural signals through a thinned skull with μECoG recording grids may provide a useful balance between invasiveness, information content, and day to day stability that could be important for future neuroprosthetics applications. In addition, this novel method may be critically enabling for neuroscience studies in which minimizing the trauma to the underlying neural or non-neuronal cells of interest is necessary to avoid potential confounds given the fundamental hypothesis to be tested.

## Data Availability Statement

The datasets generated for this study are available on request to the corresponding author.

## Ethics Statement

All animal procedures were approved by the Institutional Animal Care and Use Committee (IACUC) at the University of Wisconsin–Madison, Madison, WI, United States. All efforts were made to minimize animal discomfort.

## Author Contributions

SB, JPN, and TR: experimental design, data analysis, and manuscript preparation. AS, KL, and JW: experimental design and manuscript preparation. KC, MH, and JN: data collection and data analysis. ST: device fabrication and data collection. LK-H: manuscript preparation. All authors contributed to draft the manuscript and have read and approved the final manuscript.

## Conflict of Interest

JW and KL are scientific board members and have stock interests in NeuroOne Medical Inc., a company developing next generation epilepsy monitoring devices. JW also has an equity interest in NeuroNexus Technology Inc., a company that supplies electrophysiology equipment and multichannel probes to the neuroscience research community. KL is a co-founder and has an equity interest in Neuronoff, Inc. KL is also a paid member of the scientific advisory board of Cala Health, Blackfynn, and Battelle, and a paid consultant for Galvani. Outside of NeuroOne and NeuroNexus where the potential conflict is described in more detail here for transparency, none of these companies at present is developing technology that overlaps with the data discussed in this manuscript. The remaining authors declare that the research was conducted in the absence of any commercial or financial relationships that could be construed as a potential conflict of interest.

## Supplementary Material

FIGURE S1 Somatosensory evoked potentials (SSEPs) recorded on week 5 post-implantation from a rat implanted with a 16-channel μECoG array placed epidurally over right sensorimotor cortex. Biphasic current pulses (1 ms, varied amplitude) were used to stimulate the left hindlimb with surface electrodes over the sciatic nerve. **(A)** Stainless-steel bone screw, **(B)** Common average, and **(C)** small Laplacian referencing strategies are shown to increase the signal-to-noise ratio, and to reveal spatial signaling from the predicted hindlimb anatomical region. Dashed lines represent onset of electrical stimulus.

FIGURE S2 Line plots of individual impedance values recorded at 1 kHz of rats implanted with μECoG arrays over **(A)** thinned skull and implanted **(B)** epidurally. Each color represents an individual animal. Data shown here is from the same animals as used in Figure 5 main text.

FIGURE S3 Immunohistology of astrocytes and microglia under a μECoG thinned skull implant (left hemisphere) and μECoG epidural implant (right hemisphere) after 1 month of implantation in the same rat. **(A)** GFAP (in green) shows astrocyte distribution and boxes indicate where the μECoG arrays were placed. **(B)** Astrocyte densities comparable to the area outside the implant are seen under a thinned skull implant. **(C)** Possible increased astrocyte densities and elongated processes are seen in the area beneath the epidural implant. **(D)** IBA-1 staining (in red) labeled microglia distribution. **(E)** Microglia staining comparable to the area outside the implant are seen under a thinned skull implant. **(F)** Possible increased microglia densities and thickened dura can be seen on the epidural implant hemisphere.
