# Supplementary figures and images for: μECoG Recordings Through a Thinned Skull
Source: Front Neurosci. 2019 Oct 1;13:1017. doi: 10.3389/fnins.2019.01017 (PMC6779785; doi:10.3389/fnins.2019.01017)

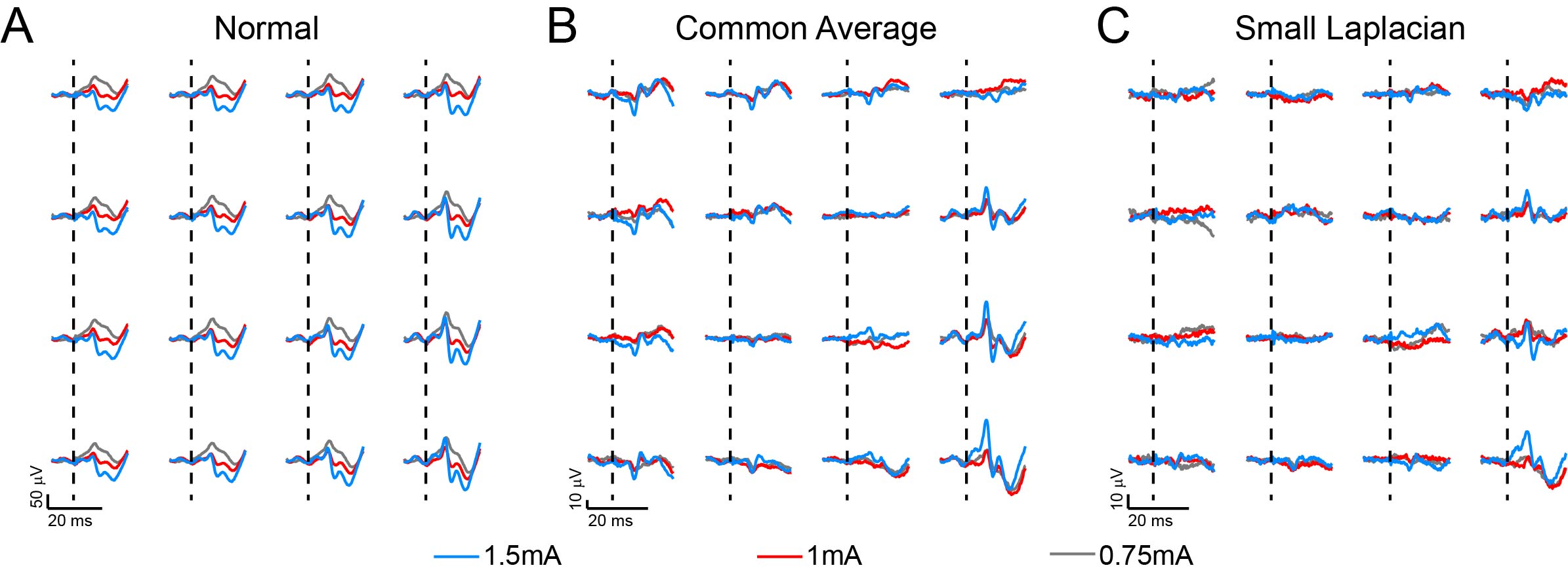

Supplement: FIGURE S1 — Somatosensory evoked potentials (SSEPs) recorded on week 5 post-implantation from a rat implanted with a 16-channel μECoG array placed epidurally over right sensorimotor cortex. Biphasic current pulses (1 ms, varied amplitude) were used to stimulate the left hindlimb with surface electrodes over the sciatic nerve. (A) Stainless-steel bone screw, (B) Common average, and (C) small Laplacian referencing strategies are shown to increase the signal-to-noise ratio, and to reveal spatial signaling from the predicted hindlimb anatomical region. Dashed lines represent onset of electrical stimulus. [file Image_1.jpg]

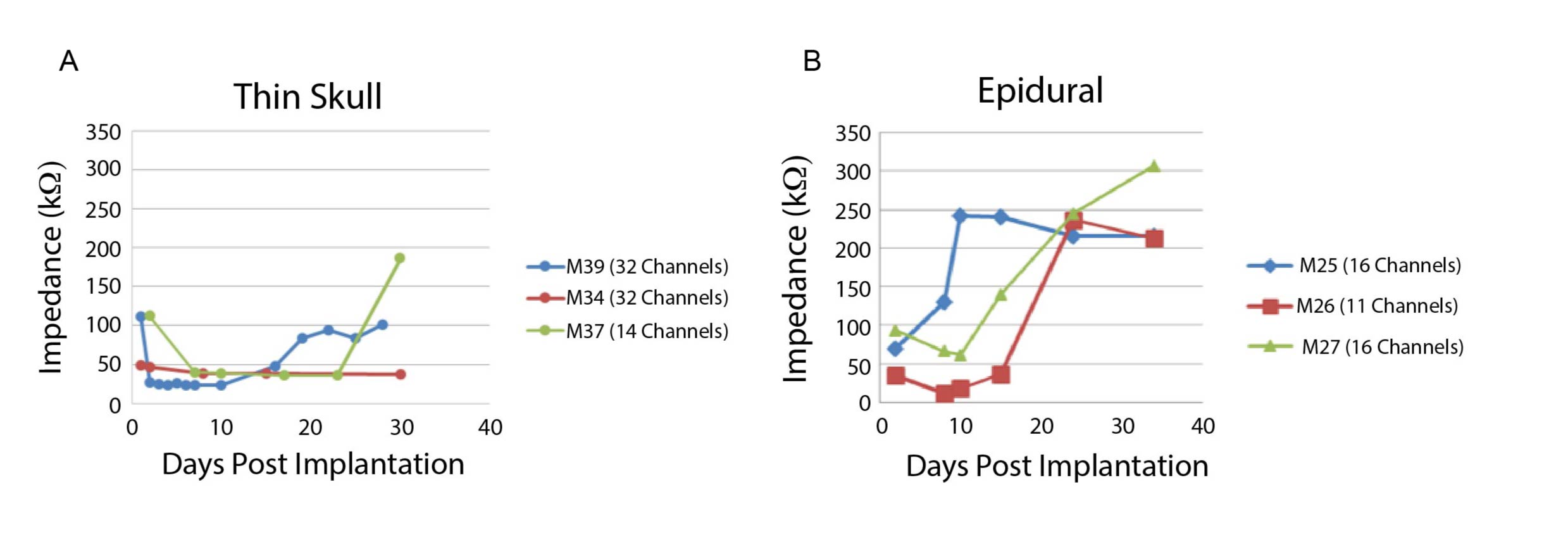

Supplement: FIGURE S2 — Line plots of individual impedance values recorded at 1 kHz of rats implanted with μECoG arrays over (A) thinned skull and implanted (B) epidurally. Each color represents an individual animal. Data shown here is from the same animals as used in Figure 5 main text. [file Image_2.jpg]

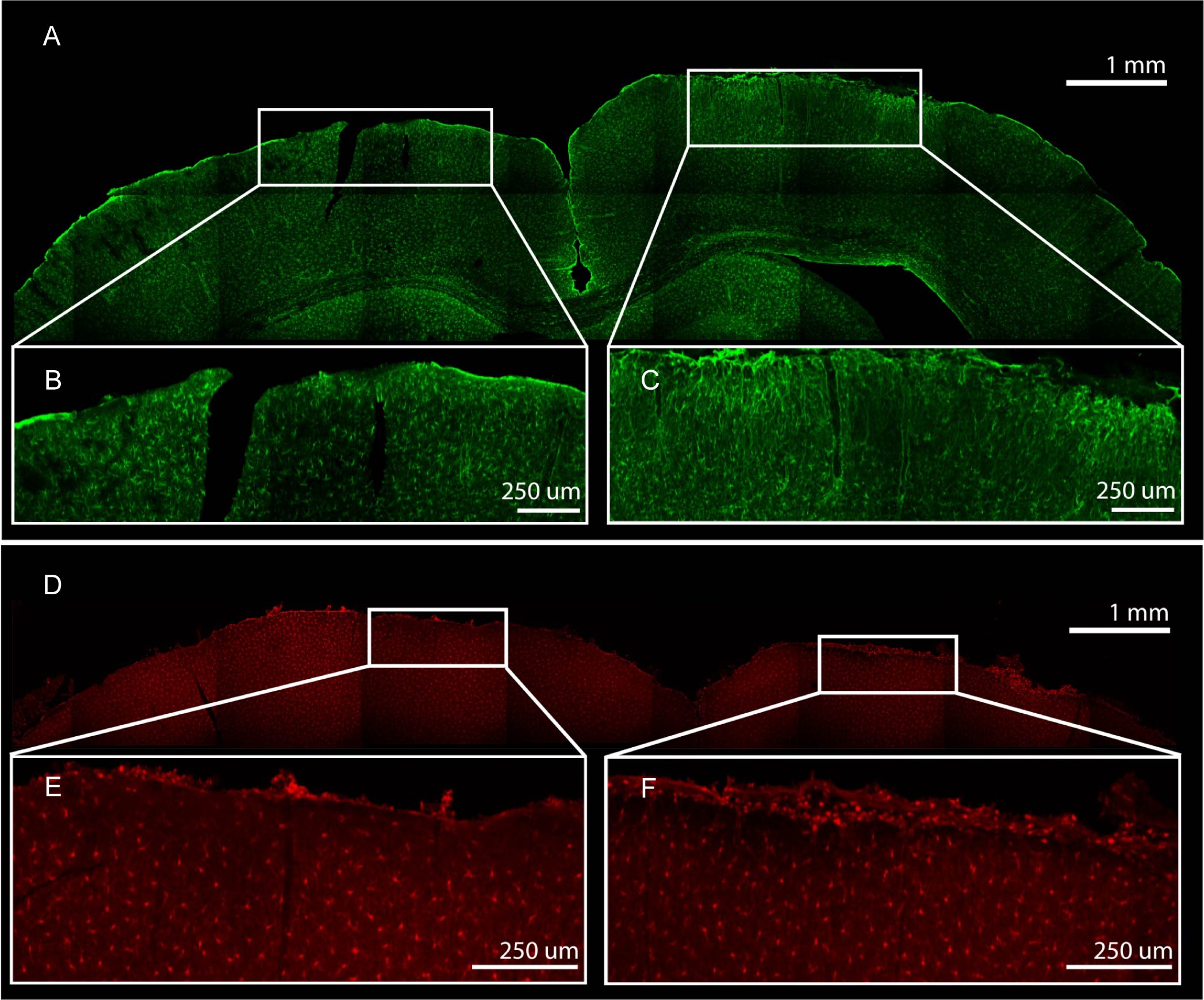

Supplement: FIGURE S3 — Immunohistology of astrocytes and microglia under a μECoG thinned skull implant (left hemisphere) and μECoG epidural implant (right hemisphere) after 1 month of implantation in the same rat. (A) GFAP (in green) shows astrocyte distribution and boxes indicate where the μECoG arrays were placed. (B) Astrocyte densities comparable to the area outside the implant are seen under a thinned skull implant. (C) Possible increased astrocyte densities and elongated processes are seen in the area beneath the epidural implant. (D) IBA-1 staining (in red) labeled microglia distribution. (E) Microglia staining comparable to the area outside the implant are seen under a thinned skull implant. (F) Possible increased microglia densities and thickened dura can be seen on the epidural implant hemisphere. [file Image_3.jpg]
